# Deep Spatio-Temporal Memory and Interaction Network for Traffic Incident Detection

**DOI:** 10.3390/s26154979

**Published:** 2026-08-06

**Authors:** Wei Hu, Yuxing Zhang, Hongjun Li

**Affiliations:** School of Information Science and Technology, Nantong University, Nantong 226019, China; zhangyuxingntu@163.com (Y.Z.); lihongjun@ntu.edu.cn (H.L.)

**Keywords:** traffic incident detection, spatio-temporal information perception, temporal feature learning

## Abstract

In traffic scenes, identifying traffic incidents captured by onboard cameras in driving videos is an essential task. Traffic incidents in driving videos usually have short durations, and the background dynamics are complex, making traffic incident detection a challenging task. To better detect traffic incidents in driving videos, this paper proposes a traffic incident detection method based on a deep spatio-temporal memory and interaction network. First, in this study, a spatio-temporal information perception network is designed that can effectively capture the core semantics from small spatial areas to large spatial areas, as well as short-term features in the temporal domain, to enhance the video frame feature extraction ability and provide a more accurate classification for traffic incidents. Second, this paper proposes a temporal feature learning network so that the spatio-temporal information perception network can fully capture long-term temporal information when processing video frames. This method enhances the learning of time features to obtain more complete contextual temporal information features and improve the accuracy of determining the abnormal occurrence and termination times. To evaluate our method, we conducted extensive experiments on the DoTA dataset containing 4677 videos with temporal, spatial, and categorical annotations. Our experimental results show that our method achieves an AUC of 80.9% for traffic scenes. These results are highly competitive when compared with the existing state-of-the-art methods, demonstrating good traffic incident detection classification accuracy and efficiency.

## 1. Introduction

Autonomous vehicles still face significant challenges in accident detection and prediction, a problem that has received extensive attention in both industry and academia. In recent years, the popularity of dashboard cameras has made them an important tool for recording accident scenes. This unique advantage not only improves the recording ability of accident scene data, but also provides richer sample data for the training of deep learning models. Dashcam-based models will become more reliable in the future, thus significantly improving the decision-making ability of autonomous vehicles in accident scenarios.

However, there is a clear difference between the video captured by the dashcam and the data characteristics of highway or traffic light surveillance cameras. The dashcam records traffic videos in a horizontal view, where both the camera and surrounding objects are moving, which increases the complexity of traffic incident detection, especially when it involves classifying objects and traffic participants approaching the dashcam. Therefore, it is important to identify traffic incidents, including anomalies of vehicles and pedestrians, in natural driving videos captured by onboard cameras. This task is not only applicable to road safety warning [1,2], autonomous driving [3], and traffic flow control [4], but also can effectively promote pedestrian protection [5]. Although many researchers have worked on anomaly detection methods under static surveillance in recent years [6,7], these methods often struggle to achieve ideal traffic incident detection performance due to the dynamic and complex background of driving videos [8].

The model utilized for traffic incident detection in traffic recorders usually adopts a hybrid deep learning framework, which combines a convolutional neural network (CNN) and a recurrent neural network (RNN). In this study, we aim to identify the potential hazards in dashcam videos. These methods utilize CNNs to perform feature extraction and object localization on images, and the extracted spatial features are subsequently fed into RNNs to capture temporal patterns and understand the evolution of these spatial features over time. Mask R-CNN was used for semantic segmentation; then, the convolutional neural network–long short-term memory (CNN-LSTM) network was used to detect and classify dangerous lane changes based on Mask frame coverage [9]. However, unguided deep learning models may generate spurious patterns [10], and CNN models also have limitations in extracting unobservable contextual relationships [11]. In recent research, articles [12,13] proposed that, in traffic incident detection, the main challenge lies in solving the problem of class imbalance and anomaly class diversification.

Although the above methods improve the detection ability to some extent, further research is needed to combine the spatio-temporal information of traffic videos. Therefore, it is still an urgent challenge to design a model that can simultaneously consider the difference in the movement of the vehicle recorder and the scale change characteristics of the target in the video, and combine the spatial characteristics and long-term and short-term temporal information in the video frame to quickly detect and accurately determine the onset time of the traffic incident.

To address the aforementioned limitations of existing multi-level spatio-temporal models, this work proposes a deep spatio-temporal memory and interaction network (DSTMIN) for first-person traffic video analysis. Unlike prior frameworks that rely on rigid downsampling and fixed convolutions, the DSTMIN extracts hierarchical local-to-global spatio-temporal features via two dedicated cascaded stages, achieving fine-grained real-time traffic incident description without using any future information. In the first spatio-temporal perception stage, MorphFCs are introduced along the spatial dimension to mine scene semantic information, while MorphFCt on the temporal channel captures frame-wise feature variations [14]. This design eliminates the redundant unfolding, reshaping, and folding operations required by the sliding-window paradigm in the Video Swin Transformer, and embedded residual connections between MorphFC inputs and outputs further stabilize training for more effective extraction of spatial details and short-term temporal dependencies. Built on the short-term features from the first stage, the second temporal context learning stage adopts a dual-branch dilated convolution residual structure. It expands the receptive field without any extra computational cost or resolution loss, capturing long-range temporal contexts that conventional models fail to access, to accurately determine the exact onset time of traffic incidents. The proposed method can be mainly divided into the following two parts:

(1) This paper proposes a spatio-temporal information perception network, which can effectively capture the local-to-global information in the spatial domain and short-term features in the temporal domain from the input video frames, and improve the extraction ability of video frame features. This facilitates more accurate anomaly classifications in subsequent traffic video analyses.
(2) We design a temporal feature learning network, which aims to complement the inter-frame dependencies learned by the existing spatio-temporal information perception networks by aggregating temporal markers at each spatial location. Considering that there is a large amount of local and global long-term temporal information between video frames, the proposed network can effectively process this information to achieve more comprehensive temporal feature extraction, which helps to improve the ability to accurately determine the onset time of anomalies.

## 2. Related Works

The traffic incident detection studied in this paper implements anomaly detection in the context of dashcam video, which is widely used in various fields to identify deviations from regular patterns. This detection involves identifying simple visual anomalies, such as the sudden motion of a specific region in a video frame, or the presence of visual artifacts or objects not included in the training datasets. Unlike the simple visual anomaly perspective, driving scenes are considered to be continuously moving, and anomalies are usually defined by complex interactions between traffic participants, which indicate a clear imbalance between normal driving scenes and accident scenes. To address these challenges, researchers have proposed a variety of correlation detection methods, which can be divided into three main types: video-based, segment-based, and frame-based detection methods.

Video-based traffic incident detection methods consider the whole video as the input of traffic anomaly detection (TAD) models. These methods are usually divided into traditional/classical classifiers and reconstruction error-based models. While traditional classifiers operate under the assumption that all normal instances belong to a single class, reconstruction error-based models rely on the premise that abnormal samples exhibit higher reconstruction errors when compared to normal samples. As described in previous work [15,16,17], one-class classification methods aim to construct compact multidimensional representations for normal data. During the testing phase, any data point that falls outside the learned multidimensional representation is labeled as an anomaly. The disadvantage of this classifier is that features need to be extracted separately [18]. To achieve end-to-end video analysis, several studies have proposed reconstruction error-based models, which extract feature maps from input videos and distinguish accident/abnormal videos from normal ones (small errors) using reconstruction errors (large errors). These methods are further subdivided into generative adversarial network (GAN)-based methods [19] and autoencoder-based networks [20]. Nonetheless, facing the uneven distribution and dynamic and complex background of dashcam accident videos, video-based methods still cannot meet the requirements of TAD.

Segment-based traffic incident detection aims to solve the problem of unbalanced distribution in traffic incident detection. Such techniques usually adopt a weakly supervised framework combined with the multiple instance learning principle. For example, in some studies [21,22,23], videos are divided into multiple segments, where abnormal videos may be intermingled with normal segments before and after abnormal segments. We use anomaly and normal class labels for the whole video rather than for each video clip, aiming to directly learn anomaly scores for each video clip, thereby reducing manual annotation time. Video clips are first fed into the MIL model to extract features, and a 3D convolutional network is subsequently applied [23]. Each segment is assigned an anomaly score according to which it is judged to be abnormal or normal. In addition, article [22] introduces the optical flow of each segment as a feature and focuses on the MIL method to improve the overall detection performance. Article [24] proposed a more complex two-branch network structure consisting of a spatio-temporal dynamic model and an interconnection dynamic model, both of which were trained using MIL techniques. The former inputs video clips, while the latter inputs feature maps describing the interaction between pedestrians and the environment. Furthermore, article [25] introduces the location of anomalies into the segment-based detection methods. Despite the potential of segment-based methods for TAD and localization, they still have limitations when dealing with multiple anomalies in complex scenes.

Frame-level traffic incident detection methods, such as those described in articles [26,27], detect the reconstruction difference between the generated current frame and the original frame by constructing the relationship between the current frame and the previous frame. Although this method can accurately determine the spatial and temporal location of abnormal events, it is computationally expensive and similar to video-based and segment-based methods. Such methods can also be divided into two categories: classifier-based methods and reconstruction error-based methods; the former usually adopts the strategy of supervised learning [28]. Autoencoder networks [29,30,31,32] generate approximate future frames after extracting key features by compressing input frames into “bottlenecks” or latent vectors. However, autoencoders are vulnerable to overfitting and vanishing gradient problems, which are common challenges in neural networks [33]. Contrarily, a GAN [34] aims to generate synthetic data that is highly similar to real data through adversarial learning between the generator and the discriminator. To address the anomaly detection problem, article [35] proposed AnoPred, which leverages multi-task loss including image intensity, optical flow, gradient, and adversarial loss, aiming to achieve video anomaly detection. However, AnoPred is designed primarily for video surveillance environments, where the prediction of abnormal traffic incidents is difficult due to its dynamic nature in videos captured by dashboard-mounted cameras. In article [13], researchers designed an STFE model with a two-stage detector: first, the optical flow histogram [36] and the ordinal CNN features of the frame were used for rough detection, then the CNN features and the spatial relationship of objects were further refined by encoding. In addition, the FOL method in article [37] focuses on tracking the position of the actor, rather than predicting the future of the whole frame. The applicability of both methods is limited by the presence of pedestrians in the field of view. The AnoPred [35], STFE [13], and FOL [37] methods all rely on additional input information other than RGB frames, such as the optical flow of the moving camera, bounding boxes, and ego-motion information. Finally, the TRN model described in article [38] combines the action detection task with the time dependence of RNN modeling, and it deals with future expectations through the time decoder. In contrast, the proposed model focuses on processing RGB frames, thus highlighting the ability to understand traffic incidents in traffic scene videos without auxiliary information.

Traffic incident detection has advanced rapidly along some key technical lines. Transformer-based methods [39] have excelled at modeling long-range spatio-temporal dependencies, with recent dual-stream and temporal-enhanced variants delivering consistent performance gains on mainstream benchmarks. Meanwhile, large-scale vision foundation models [40] have reshaped the field by introducing cross-modal semantic alignment and open-set recognition capabilities, pushing the state of the art far beyond the limits of traditional closed-set traffic incident detection systems.

## 3. Methodology

**Figure 1:**
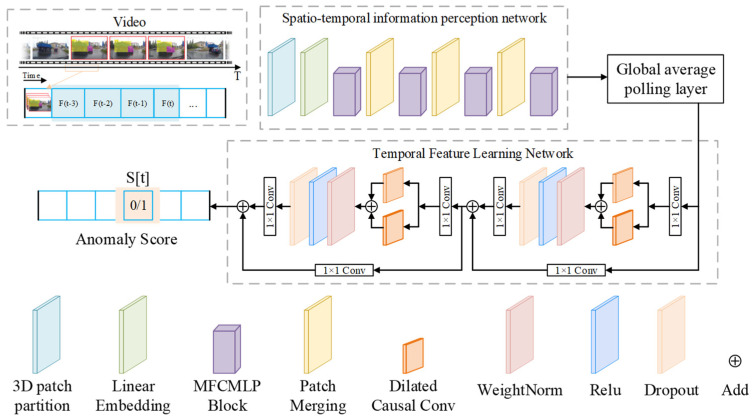
Deep spatio-temporal memory and interaction networks.

In traffic scenes captured from a driver’s first-person perspective, the size of obstacles and traffic incident targets changes with distance, which poses challenges for the accurate detection and classification of such events. To improve the detection performance, this paper proposes a traffic incident detection method based on a DSTMIN, as show in in Figure 1. The model is mainly composed of two parts: a spatio-temporal information perception network and a temporal feature learning network. In the spatio-temporal information perception network, MorphFCs is integrated into the spatial dimension to extract spatial semantic information, and MorphFCt is used to model the relationship between video features across time in the temporal dimension. These temporal features are then remapped to the spatial dimension, thus enhancing the ability to capture spatial and short-term temporal information from video frames. The temporal feature learning network focuses on the effective extraction of long-term temporal information. Since short-term temporal features are already extracted at the initial stage, the network employs a two-branch residual structure and dilated convolution to better capture long-range temporal dependencies. This structure helps to improve the detection of traffic incidents over extended time intervals. Finally, the model integrates local and global spatio-temporal information features, which can locate traffic incidents more accurately in time and classify traffic incident types accurately. The proposed DSTMIN method significantly enhances the ability of the model to detect and classify traffic incidents in complex dynamic driving scenarios.

### 3.1. Spatio-Temporal Information Perception Network

**Figure 2:**
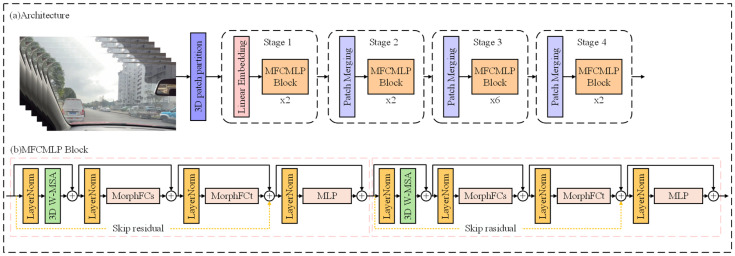
The framework of spatio-temporal information perception network.

To better deal with the spatio-temporal features in traffic videos, this paper proposes a spatio-temporal information perception network (STIPN), which can effectively capture the spatial and temporal domain features in video frames. Specifically, after preprocessing the input video frame, the network handles different scales by dividing the resulting token sequence into blocks, and it gradually captures the local-to-global spatial and temporal information by gradually increasing the block length. This processing method can extend the local information to the global information in the spatial domain, and it can gradually capture the information characteristics in the time domain. Through this multi-scale spatio-temporal perception, the network can extract the key features in the video frames more comprehensively, so as to classify the abnormal events, accurately determine the abnormal time in the subsequent traffic video anomaly detection, and achieve better detection results. The overall framework is shown in Figure 2.

**Figure 3:**
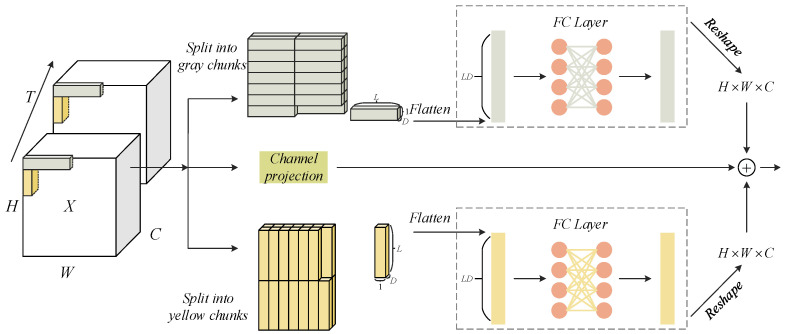
MarphFCs in the spatial dimension, L scales hierarchically with the depth of the network.

In traffic video processing, mining the core spatio-temporal semantic information is crucial for the recognition and classification of traffic incidents in the video. Currently, the typical CNN and MLP only focus on the modeling of local or global information; thus, they cannot effectively capture the local-to-global information in the video sequence. To solve this problem, in this study, the MFCMLP module is designed, in which MarphFCs is first introduced, which can hierarchically expand the receptive field of FC and make it operate from a small area to a large area. The referenced MarphFCs processes each frame of video independently in horizontal and vertical paths. As shown in the following Figure 3, this study mainly considers a horizontal block as an example (the green block in Figure 3).

Specifically, given an input video frame $X\in R^{HW\times C}$, which is projected into a token sequence, this study first splits X along the horizontal direction and sets the block length to L, which can be obtained as follows:

(1) $$X_{i}\in R^{L\times C}$$

where $i\in \left\{1,\ldots ,HW/L\right\}$. To reduce the computational cost, in this study, each *X_i_* is divided into multiple groups along the channel dimension, where each group has D channels. Thus, the segmentation blocks can be obtained; each block is

(2) $$X_{i}^{k}\in R^{L\;D}$$

where $k\in \left\{1,\ldots ,C/D\right\}$. Next, each data block is flattened into 1D vectors, and the FC weight matrix $W\in R^{L\;D\times LD}$ is applied to each data block for rotation; that is, Y is

(3) $$Y_{i}^{k}\in X_{i}^{k}W$$

After the feature transformation, all blocks $Y_{i}^{k}$ are reshaped to their original dimensions $Y\in R^{H\times W\times C}$. The same is true for the vertical direction, except that the marker sequence is split along the vertical direction. To allow communication between groups along the channel dimension, FC layers are also applied to process each token individually. Finally, the lateral, vertical, and channel features are summed element-wise to obtain the output result. As the network goes deeper, the block length L increases hierarchically, thus enabling the FC filter to gradually discover more core semantics from small to large spatial regions.

**Figure 4:**
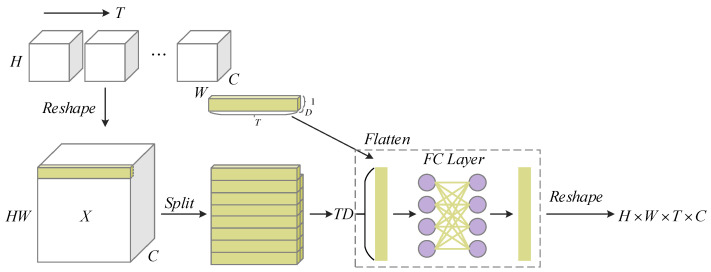
MarphFCt acting on the temporal dimension.

When processing traffic videos, it is necessary to process not only the spatial horizontal and vertical path information in consecutive video frames but also the temporal channel information. The MLP in VST usually operates independently on a position-by-position basis; that is, the feature vector at each position is linearly transformed through the fully connected layer independently. Thus, the relevance between different locations and the dependencies in the time dimension cannot be directly captured. Therefore, we also introduce MorphFCt into MFCMLP, which transforms the input feature map in the time dimension and flattens, fully joins, and restores it to the high-dimensional space. Such processing can be used to capture the relationship of the input video features over time and to remap these temporal features to the spatial dimension. The principle and network architecture are shown in Figure 4.

Assuming an input video clip labeling $X\in R^{H\times W\times T\times C}$, *X* is first divided into groups (D channels per group) along the channel dimension to reduce the computational cost, yielding

(4) $$X^{k}\in R^{H\times W\times T\times D}$$

where $k\in \left\{1,\ldots ,C/D\right\}$. For each spatial location s, this study concatenates the features of all frames into a block $X_{s}^{k}\in R^{TD}$, where $s\in \left\{1,\ldots ,HW\right\}$.

Next, this study uses the FC matrix $W\in R^{TD\times TD}$ to transform the temporal features and to obtain the following:

(5) $$Y_{s}^{k}\in X_{s}^{k}W$$

Finally, this paper rescales all the blocks back to the original token dimension and outputs $Y\in R^{H\times W\times T\times C}$. In this way, the FC filter can simply aggregate the token relationships within the block along the time dimension, providing VST with stronger modeling ability in the time dimension. At the same time, the residual connection between the original input and output of MarphFCs is adopted to increase the stability of model training.

### 3.2. Temporal Feature Learning Network

**Figure 5:**
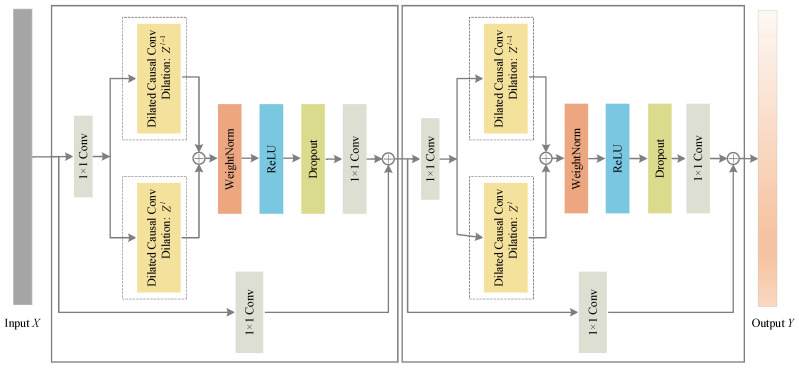
The framework of temporal feature learning network.

The STIPN can learn the dependence on the already input frames by aggregating the temporal markers at each spatial location. However, there is much local and global temporal information between video frames that needs to be processed to obtain more complete temporal features, so as to determine the anomaly time more accurately. In this paper, a temporal feature learning network (TFLN) (shown in Figure 5) is proposed to improve the ability to learn the temporal features in the processed feature map. In Figure 5, the 1 × 1 convolution operation only considers the temporal information on neighboring frames, and this operation can effectively capture the short-term dependencies between video frames and extract local temporal features. Secondly, this study uses the parallel processing of dilated convolution with a dilation rate of 3 in the time domain. This parameter enables the convolution kernel of the same size to obtain a larger receptive field without increasing the computational complexity. At the same time, in this study, two series structures are designed to form a TFLN, which supplements the time information at the interval missed by the input time series under the action of the dilated convolution with an expansion rate of 3. Through such a design, even the use of a smaller convolution kernel can capture the features of a longer time range, so as to process the global temporal information. In this way, information can be gathered from more distant time frames without losing local details.

In the input time series, it is assumed that the input time series is ${\hat{X}}^{\left(i-1\right)}=({\hat{X}}_{1}^{\left(i-1\right)},\;\ldots ,\;{\hat{X}}_{T}^{\left(i-1\right)})$, where T denotes the time step, obtained after the convolutional transform of

(6) $$Z=W_{1}{\hat{X}}^{\left(i-1\right)}+b_{1}$$

The parallel processing part of the cavity convolution mainly consists of two parallel convolutional processing blocks, each of which contains two dilated convolutional layers; for each dilated convolutional layer, the output of the layer l can be expressed as follows:

(7) $$X^{\left(l\right)}=Dilated\;Conv(X^{\left(l-1\right)},\;dilation=d_{l})$$

where $d_{l}$ refers to the expansion rate of the layer l.

Assuming that the output of the first dilation convolution block is $X_{1}$ and the output of the second dilation convolution block is $X_{2}$, the specific processing of each block is as follows:

(8) $$X_{1}=Dilated\;Conv_{1}(W_{1}Z+b_{1})$$

(9) $$X_{2}=Dilated\;Conv_{2}(W_{2}Z+b_{2})$$

Then, the information obtained after residual concatenation and subsequent processing is as follows:

(10) $$X=X_{1}+X_{2}$$

After a series of nonlinear activation and regularization, we obtain the following:

(11) $$X_{\mathrm{norm}}=WeightNorm(X)$$

(12) $$X_{relu}=Relu(X)$$

(13) $$X_{dropout}=Dropout(X)$$

The final output is as follows:

(14) $$Y=W_{3}Dropout(Relu(WeightNorm(\;X_{1}+X_{2})))+\;b_{3}$$

The TFLN processes local and global temporal information in videos through inflationary and causal convolution and achieves the comprehensive modeling of temporal information in video frames through multi-branch and multi-scale feature fusion.

For each video frame F[t], the final output anomaly score S[t] ∈ [0, 1], where 0 indicates no anomaly, and 1 indicates that the frame is abnormal. To reflect the higher weight given to the anomaly class of the data distribution, in this study, the weighted cross-entropy loss is chosen to train the model. The formula for each weight $\omega_{i}$ is as follows:

(15) $$\omega_{i}=e/e_{i}$$

where e denotes the total number of examples in the dataset, and $e_{i}$ denotes the number of examples in category i.

## 4. Experiments

### 4.1. Dataset Introduction

In article [37], a new publicly available dataset of driving video anomalies in traffic scenes, named DoTA, was proposed for traffic incident detection and recognition. It consists of 6000 video clips mainly from two YouTube channels that provide traffic accident videos for driver education purposes. We selected diverse dash camera accident videos from different areas and lighting conditions. We did not select videos with accidents that were not visible or where the camera dislodged during the accident, yielding 4677 videos with 1280 × 720 resolution, each containing exactly one anomalous event. We randomly partitioned DoTA into 3275 training and 1402 test videos and used these splits for all tasks.

In DoTA, each DoTA video is annotated with an abnormal start and end time. It is divided into three time zones: the prephase, which is the normal video before the anomaly, the time window of the anomaly, and the normal activity after the anomaly. As early detection is crucial for road anomalies, gaze estimates the anomaly start time as the time at which the anomaly is inevitable. The end time of the anomaly is approximated by the time when all the anomalies are physically out of view or stationary. DoTA is the first traffic video dataset to provide the detailed spatio-temporal annotations of anomalous objects. The dataset provides each anomalous object with a unique trace ID and a label for its bounding box. In addition, each DoTA video was assigned to one of the nine categories listed in Table 1. Therefore, in this study, the DoTA dataset is adopted in the experiments evaluating the performance of different methods.

### 4.2. Evaluation Index

Based on the existing research, this study uses the area under the receiver operating characteristic (ROC) curve (AUC) and accuracy as the evaluation metrics. The relationship between true positive rate (TPR) and false positive rate (FPR) was plotted by changing the threshold of the abnormal score, and the ROC curve was obtained. The AUC value was mainly used to evaluate the robustness and efficiency of the model. When the AUC value was higher, the model could better distinguish normal and abnormal events. To ensure comparability between different methods, the AUC of frame-level detection is calculated in this study. AUC can be calculated using the FPR as the integral of the TPR values:

(16) $$AUC=\int_{0}^{1}TPR(x)dx$$

### 4.3. Implementation Details

The proposed model is trained and evaluated on an Nvidia GeForce RTX 3090 server, and Pytorch 1.11.0 is used for experiments. Secondly, to make the training of the model more stable, this paper uses the SDG optimizer for training, with a batch size of 4, a learning rate of 0.00005, a momentum of 0.9, and a video clip length of 8. To train the network, the size of the input video frames is set to 640 × 480, the dataset is split according to the official protocol, and the training period for the DoTA dataset is set to 100 epochs. In this study, the weighted cross-entropy loss is used for training to solve the problem of unbalanced data in the DoTA dataset, $\omega_{n}$= 0.3 and $\omega_{a}$= 0.7 for the normal and abnormal classes, respectively, and $\omega_{i}=e/e_{i}$.

### 4.4. Comparison with Existing State-of-the-Art Techniques

In this study, the proposed traffic incident detection method based on a DSTMIN is compared with existing related anomaly detection methods, including LSTM [41], ConvAE [42], ConvLSTMAE [43], TAD [21], TAD + ML [44], FC [44], Encoder–Decoder [45], AnoPred [35], FOL-Ensemble [37], Movad [39], TRN [37], RTFM + M3 [40], and STFE [13]. Among these methods, FC is a video-level detection method; TAD, TAD + ML, and Ensemble are segment-level detection methods based on localization; ConvAE, ConvLSTMAE, AnoPred, LSTM, Encoder–Decoder, Movad, TRN, and STFE are frame-level detection methods. Table 2 shows the AUC results of the different methods on the DoTA dataset.

It is evident from Table 2 that the proposed method achieves an AUC of 80.9%, which is better than that of all other methods. Contrarily, the AUC of the video-level detection method FC is 61.7%, and its accuracy is low. As this method deals with whole videos rather than fine-grained segments or frames, it exhibits limitations in capturing details. Location-based segment-level methods such as TAD and TAD + ML achieve AUCs of 69.2% and 69.7%, respectively, which are slightly improved when compared with video-level methods, because segment-level detection can determine the time period of the event more accurately. However, since these methods are still processed at the segment level, some important details may be missed. Frame-level detection methods include LSTM, ConvAE, ConvLSTMAE, AnoPred, Encoder–Decoder, Movad, TRN, and STFE. They capture more detailed temporal information using frame-by-frame analysis; thus, their performance is generally better than that of video-level and segment-level methods. Among them, STFE performs better in frame-level detection with an AUC of 79.3%. Therefore, the AUC performance of the method proposed in this paper is significantly better than that of other methods. The model extracts the local-to-global spatio-temporal information in traffic videos using two different networks, so as to more accurately detect the category of traffic incidents and determine the start time of traffic incidents.

As can be seen in Table 3, there are obvious differences in the AUC performance of different methods in various types of traffic incident classification tasks. When compared with other methods, the proposed method achieves the best AUC scores in most traffic incident categories. There are fluctuations in the performance of methods such as AnoPred, FOL-STD, and FOL-Ensemble in different categories. Especially in complex traffic incident types, such as the OO category, the AUC scores of these methods are generally low. The proposed method exhibits high performance in complex accidents, which further demonstrates the robustness of the model. When compared with STFE, the proposed method performs better in most scenarios (such as ST, LA, OC, TC, VP, VO, OO, and UK), especially in ST and OO traffic incident detection tasks. However, the detection performance in the AH category is slightly lower, which may be because the target obstacles in the driving video are too small or some are suddenly flying abandoned objects. The method proposed in this paper cannot fully handle the detection of such traffic incidents, but its overall performance is beyond satisfactory. In Table 3, the proposed method exhibits optimal performance in ST, AH, LA, OC, TC, VP, and other categories; especially in ST, LA, OC, VP, and other categories, its performance is significantly high. As shown in Table 4, the proposed method performs well in the categories of *ST, *AH, *LA, *OC, *TC, *VP, *VO, *OO, and *UK in the classification of traffic incidents with non-self anomalies. In general, based on the DSTMIN, the proposed method achieves the highest AUC in almost all traffic incident categories, especially in the key categories such as ST, LA, OC, TC, VP, VO, OO, and UK, demonstrating a high classification accuracy. The proposed method achieves the highest AUC value in most categories, and it has good robustness and generalization ability.

### 4.5. Visual Analysis

**Figure 6:**
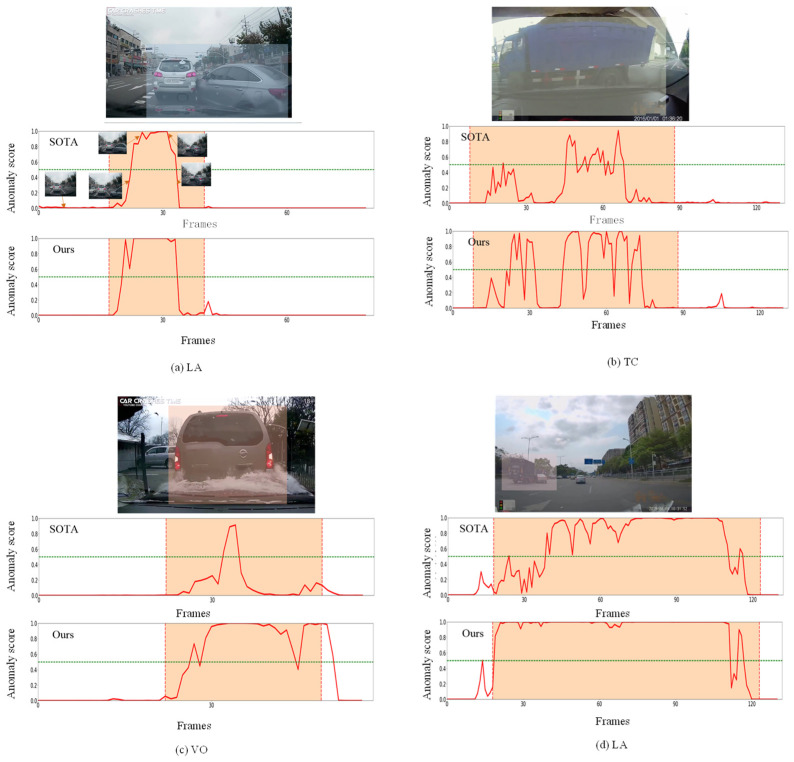
Visual comparison of traffic incident detection examples from the DoTA dataset.

It is evident from Figure 6a that the anomaly score tends to 0 when the car is normally driven on the road. The anomaly score rises significantly when there is a deviation in the car’s driving on the road. When a collision occurs, the anomaly score is close to 1. This indicates that the method proposed in this paper can detect an anomaly and can determine the time when the anomaly starts and ends. Figure 6 presents the visualization examples of traffic incident detection for the self-abnormal category and the non-abnormal-self category, and it compares and analyzes the visualization result graph of STFE(SOTA) [13]. Within the anomaly region in Figure 6a, the anomaly score of the SOTA method rises slowly and decreases rapidly after the peak position is briefly maintained, showing a certain degree of volatility. In contrast, the anomaly score of the proposed method reaches 1 more quickly and remains stable in the abnormal region, and the overall fluctuation is smaller. In Figure 6b, the anomaly score of the SOTA method fluctuates significantly in the abnormal region, containing multiple small peaks, reflecting the inaccuracy of the detection. However, the anomaly scores obtained by the proposed method remain relatively stable in the abnormal region, showing a relatively stable high value overall, although there are also some fluctuations. In Figure 6c, the anomaly score obtained by the SOTA method shows a gradual upward trend in the abnormal region, but it decreases rapidly after approaching 1, accompanied by large fluctuations. However, the anomaly score obtained by our method quickly reaches 1 and remains stable within the anomaly region without significant fluctuations. In Figure 6d, the output curve of the SOTA method fluctuates greatly in the abnormal region and fails to stably reach a high value, showing the inaccuracy in detecting this kind of traffic incident. In contrast, the anomaly scores obtained by the proposed method remain stationary in the anomaly region and are close to 1 with almost no significant fluctuations. In summary, the two-stage spatio-temporal information fusion method shows a smoother abnormal score curve in the four given scenes, showing high accuracy and stability of traffic incident detection and classification.

### 4.6. Ablation Experiments

In order to better verify the effectiveness of the two-stage spatio-temporal information combining network performance on the DoTA dataset, this study conducts ablation experiments, as shown in Table 5. Firstly, when the STIPN is used alone, the AUC of the model is 74.17%, and its accuracy is 73.04%. Second, when only the temporal features are used to learn the network, the AUC and ACC of this model are 72.08% and 71.33%, respectively. When the STIPN and the TFLN are included at the same time, the AUC and ACC of the model are 80.9% and 76.05%, respectively, and the performance based on the DSTMIN is the highest. This shows that the proposed model can extract more effective spatio-temporal information features in video frames under the joint action of STIPN and TFLN and capture the dependence of different video frames in the spatio-temporal domain, so as to better classify traffic incidents and determine the start time of traffic incidents.

### 4.7. Running Time

This experiment is carried out on an Nvidia GeForce RTX 3090. The running time of traffic incident detection, the size of the detected video frame image, and the running speed are 30 fps, 640 × 480, and 30 fps, respectively. The detection accuracy and classification of the model are enhanced.

## 5. Conclusions

In this study, in order to better detect and classify traffic incidents in driving videos and determine the start time of traffic incidents more accurately, a deep spatio-temporal memory and interaction network is proposed. Firstly, a spatio-temporal information perception network is designed to effectively enhance the extraction ability of video frame features by capturing the core semantic information from the local to the global spatial domain and combining short-term temporal features, so as to classify traffic incidents more accurately. Secondly, this paper proposes a temporal feature learning network to compensate for the lack of a spatio-temporal information perception network in capturing long-term temporal features. The network strengthened the learning of temporal features and captured more complete temporal context information, so that the model could determine the occurrence and end time of abnormal events more accurately. Finally, experiments on the DoTA dataset verify the effectiveness of the model. The results show that the AUC of the proposed method in traffic scenes reaches 80.9%. In future research, we will address the limitations of the model, including its reliance on a single dataset; the absence of external validation; potential challenges in generalizing across different cameras, weather conditions, and geographical environments; and the existing constraints in real-time practical deployment.

## Figures and Tables

**Table 1:** Traffic incidents in the DoTA dataset.

ID	Label	Category of Abnormality
1	ST	Collision with another vehicle that starts, stops, or is stationary
2	AH	Collision with another vehicle moving ahead or waiting
3	LA	Collision with another vehicle moving laterally in the same direction
4	OC	Collision with another oncoming vehicle
5	TC	Collision with another vehicle that turns into or crosses a road
6	VP	The collision between a vehicle and a pedestrian
7	VO	Collision with an obstacle in the roadway
8	OO	Out-of-control and leaving the roadway to the left or right
9	UK	Unknown

**Table 2:** AUC of different methods on the DoTA dataset (%).

Method	Type	AUC (%)
TAD [21]	Box + Flow	69.2
TAD + ML [44]	Box + Flow	69.7
FC [44]	RGB	61.7
FOL-Ensemble [37]	RGB + Box + Flow + Ego	73.0
LSTM [41]	RGB	63.7
ConvAE [42]	Gray	64.3
	Flow	66.3
ConvLSTMAE [43]	Gray	53.8
	Flow	62.5
AnoPred [35]	RGB	67.5
Encoder–Decoder [45]	RGB	73.6
TRN [37]	RGB	78.0
Movad [39]	RGB	71.8
STFE [13]	RGB	79.3
MOVAD [45]	RGB	80.1
RTFM + M3 [40]	RGB	79.8
Ours	RGB	80.9

**Table 3:** Comparison of AUC of different methods in self-abnormal traffic incident detection (%).

Model	ST	AH	LA	OC	TC	VP	VO	OO	UK
AnoPred [34]	69.9	73.6	75.2	69.7	73.5	63.3	N/A	N/A	N/A
FOL-STD [36]	67.3	77.4	71.1	68.6	69.2	65.1	N/A	N/A	N/A
FOL-Ensemble [36]	73.3	81.2	74.0	73.4	75.1	70.1	N/A	N/A	N/A
Movad [38]	75.1	71.8	70.3	72.2	73.5	70.7	73.2	69.8/73.0	65.1
STFE(SOTA) [12]	75.2	84.5	72.1	77.3	72.8	71.9	N/A	N/A	N/A
Ours	86.3	84.1	82.6	80.3	84.6	78.6	77.8	85.1/89.2	72.9

**Table 4:** Comparison of AUC of different methods in non-self abnormal traffic incident detection (%).

Model	*ST	*AH	*LA	*OC	*TC	*VP	*VO	*OO	*UK
AnoPred [34]	70.9	62.6	60.1	65.6	65.4	64.9	64.2	57.8	N/A
FOL-STD [36]	75.1	69.8	68.1	74.1	72.0	69.7	63.8	69.2	N/A
FOL-Ensemble [36]	77.5	69.8	68.1	76.7	73.9	71.2	65.2	69.6	N/A
Movad [38]	62.8	62.2	62.8	61.6	62.9	61.9	63.2	63.1/67.8	57.8
STFE(SOTA) [12]	80.6	65.6	69.9	76.5	74.2	N/A	75.6	70.5	N/A
Ours	79.5	78.5	78.4	80.2	80.4	75.4	77.7	76.1/82.2	69.6

The asterisk (*) indicates traffic incident categories involving non-self anomalies.

**Table 5:** Ablation experiments on the DoTA dataset.

STIPN	TFLN	AUC (%)	Accuracy (%)
w	w/o	74.17	73.04
w/o	w	72.08	71.33
w	w	80.9	76.05

## Data Availability

The original contributions presented in this study are included in the article. Further inquiries can be directed to the corresponding author.
